# Swallowing Function and Quality of Life in Patients Treated With Transoral Videolaryngoscopic Surgery for Pharyngolaryngeal Cancer

**DOI:** 10.7759/cureus.57143

**Published:** 2024-03-28

**Authors:** Koji Ebisumoto, Akihiro Sakai, Hiroaki Iijima, Fumiyuki Goto, Mayu Yamauchi, Daisuke Maki, Takanobu Teramura, Koichiro Wasano, Kenji Okami

**Affiliations:** 1 Otolaryngology - Head and Neck Surgery, Tokai University, Isehara, JPN

**Keywords:** hyodo score, fees, swallowing function, quality of life, transoral videolaryngoscopic surgery

## Abstract

Background: It is controversial whether transoral resection for early pharyngolaryngeal cancer preserves swallowing function and quality of life. We investigated swallowing function and quality of life before and after transoral videolaryngoscopic surgery (TOVS).

Methods: Seventy-three patients with pharyngolaryngeal cancer who underwent TOVS between July 2012 and July 2022 were enrolled in this prospective analysis. The Hyodo score and European Organization for Research and Treatment of Cancer Quality of Life Questionnaires were recorded preoperatively and at three, six, and 12 months postoperatively, in addition to the postoperative functional outcome swallowing scale (FOSS) at six months postoperatively.

Results: Although most patients could consume food orally without restrictions with a preferable FOSS score, 23 patients showed impaired Hyodo scores. Age ≥65 years significantly predicted impaired swallowing. Sub-scores of the impaired patient group showed worsening for the glottal closure reflex when the endoscope touched the epiglottis or arytenoid, as well as a reduction in the extent of pharyngeal clearance following the ingestion of blue-dyed water.

Conclusion: After TOVS, swallowing function is generally well preserved. Elderly patients, especially those with laryngeal hypoesthesia and poor clearance, are at risk of swallowing dysfunction.

## Introduction

Head and neck cancer requires multidisciplinary treatment with surgery, radiation therapy, and chemotherapy. In surgical treatment, especially extended resection, organ loss is directly related to loss of function and quality of life (QOL). However, even when chemoradiotherapy is used for organ preservation [1], serious late complications such as jaw osteonecrosis, dysphagia, persistent dry mouth, sticky saliva, and tooth decay are problematic. Organ preservation does not always mean functional preservation, which may have a rather negative impact on long-term prognosis [2,3]. Therefore, minimally invasive treatments aimed at preserving function are attracting attention and transoral resection is a promising surgical strategy. The primary surgical technique is transoral robotic surgery (TORS), which is primarily used to treat oropharyngeal cancer [4,5]. In recent years, the incidence of human papillomavirus-associated cancers has increased significantly [6], especially in developed countries, and these cancers tend to occur in the palatine and lingual tonsils compared to classic head and neck cancers. The number of TORS cases is increasing due to an increase in the number of oropharyngeal cancers [7].

TORS has only been covered by insurance in Japan since April 2022. Therefore, it has not yet gained popularity. However, efforts have focused on the early diagnosis and treatment of head and neck cancer as a double primary cancer with esophageal cancer [8]. In recent years, Japan has made notable progress in endoscopic imaging techniques, particularly with advancements like narrow-band imaging (NBI), enabling the early detection of pharyngeal cancer ahead of other countries [9]. Within this context, there has been the development of endoscopic laryngopharyngeal surgery (ELPS) as an advanced form of endoscopic esophageal cancer surgery [10]. Additionally, transoral videolaryngoscopic surgery (TOVS) has emerged, building upon traditional videolaryngoscopic surgical techniques [11]. The evolution of optical devices and surgical instruments has positioned non-robotic surgery as a crucial component of transoral surgery in Japan, recognized for its efficacy and minimally invasive nature [12-14]. Notably, at our institution, TOVS has been utilized for transoral resection of pharyngolaryngeal cancer since 2012.

While there are reports of good postoperative swallowing function in both TORS [15,16] and TOVS [17], a recently published study showed that the QOL related to swallowing function in patients treated with both TORS and radiotherapy was worse than that in patients given radiotherapy alone [18], which is controversial. Since there are few reports on the quantitative evaluation of swallowing function and QOL with transoral resection, we conducted a prospective survey on the pre- and postoperative swallowing function and QOL of patients treated with TOVS at our department.

## Materials and methods

This research adhered to the principles of the Declaration of Helsinki, and the protocol entitled “Functional evaluation for the patients with head and neck cancer and dysphagia” received approval from the institutional review board of Tokai University, number 08-R98 (approved on February 3, 2009) and 18R256 (approved on February 19, 2019). Although both studies were identical, they were reexamined at the direction of the Institutional Review Board due to the extended duration of the study. Every participant in this study gave their informed consent for inclusion.

Out of the individuals who underwent transoral TOVS for pharyngolaryngeal cancer at our department from July 2012 to July 2022, a total of 73 patients who gave their consent for participation in the prospective study were included. The inclusion criteria comprised individuals aged 18 years or older with an Eastern Cooperative Oncology Group performance status (ECOG PS) of 0. The exclusion criteria were patients younger than 18 years, ECOG PS ≥1, and those deemed inappropriate by the physician, such as those with obvious aspiration pneumonia or incapacity to consent. The eighth edition of the Union for International Cancer Control guidelines was used to classify the TNM stage.

The Functional Outcome Swallowing Scale (FOSS) [19] was recorded at six months postoperatively. The Hyodo score [20], using flexible endoscopic evaluation of swallowing (FEES) and European Organization for Research and Treatment of Cancer Quality of Life questionnaires (EORTC QLQ C30, EORTC QLQ H&N35) [21,22] was also recorded preoperatively and three, six, and 12 months postoperatively. The patients were classified into two groups according to whether their postoperative Hyodo score was impaired. To evaluate the predictors of worsening Hyodo score, logistic regression analysis was performed with the following predictor variables: age (≥65 years), preoperative body mass index (BMI, <18.5 kg/m^2^), primary lesion, T classification, with or without neck dissection, history of head and neck cancer, history of irradiation for head and neck, and history of esophageal cancer.

Surgical indication

Criteria for considering TOVS included the following: (1) presence of primary lesions in the oropharynx, hypopharynx, or supraglottis; (2) clinical presentation of T1 or T2 lesions; (3) absence of lymph node metastasis or presence of resectable cervical lymph node metastasis; (4) limited to within half circumferential extension; and (5) absence of distant metastases.

Exclusion criteria encompassed: (1) indications of suspected invasion on imaging studies, including the hyoid bone, thyroid cartilage, and cricoid cartilage; (2) invasion into deep tissue adjacent to the constrictor muscle or posterior invasion of the deep vertebral fascia; (3) ECOG PS of ≥ 2; (4) severe cardiopulmonary impairment; and (5) factors that might impede transoral resection, such as trismus.

Surgical procedure

The surgical approach followed the TOVS method outlined by Tomifuji et al. [14,17]. To create the surgical field, either the FK-WO retractor (Olympus Medical Systems, Tokyo, Japan) or the Weerda distending laryngoscope (Karl Storz, Tuttlingen, Germany) was utilized. Visualization of the surgical field employed a video endoscope with a movable tip, such as the OLYMPUS LTF-S190-5 or ENDOEYE FLEX 3D (Olympus Medical Systems). Electrocautery was performed using a Colorado microdissection needle (Stryker Japan KK, Tokyo, Japan) or a malleable electrocautery needle (KD 600, Olympus Medical Systems). Grasping forceps included STEINER grasping forceps, LARYNGOFORCERII grasping forceps (Karl Storz, Tuttlingen, Germany), and a MicroFrance Heart-Shaped grasper (Integra MicroFrance, Saint Aubin le Monial, France).

Lesion confirmation utilized Narrow-Band Imaging (NBI) systems and iodine-staining techniques. Tumors were resected en bloc with a safety margin of 5-10 mm. Fibrin glue and polyglycolic acid sheets were applied to cover mucosal defects [23]. 

Neck dissection was performed in cases with cervical lymph node metastases, while tracheostomy was not performed as a routine procedure. Postoperative radiotherapy or chemoradiotherapy was considered when adverse features were confirmed through histopathological examination after surgery.

Evaluation of Hyodo score with fiberoptic endoscopic examination of swallowing (FEES)

FEES can be performed in conjunction with a laryngeal fiberscope, which is a procedure frequently used by otolaryngologists and head and neck surgeons. The Hyodo score consists of four sub-scores: (1) the salivary pooling degree at the vallecula and piriform sinuses, (2) the glottal closure reflex induced by touching the epiglottis or arytenoid with the endoscope, (3) the location of the bolus at the swallowing reflex initiation assessed by “white-out” timing, and (4) the extent of pharyngeal clearance after blue-dyed water is swallowed. These four sub-scores are rated on a four-point scale from 0-3, and the sum of the four sub-scores is the Hyodo score [20]. The scoring criteria for each sub-score are shown in Table 1. This simple scoring system has moderate sensitivity and high specificity for predicting aspiration, which provides information on whether patients are capable of oral intake, and is considered a strong predictor of aspiration when the score exceeds 6 [20]. The FEES was performed in open-label and the bolus used for the test was liquid (blue dyed water). It was evaluated by at least two head and neck surgeons and a certified nurse in nursing care of dysphagia.

**Table 1:** Scoring criteria of the Hyodo score The Hyodo score is calculated from the sum of the four sub-scores (1)-(4). Each subscore is graded 0-3, the higher the score, the worse the function. Modified and quoted from Chiba et al. [20]

(1) The salivary pooling degree at the vallecula and piriform sinuses	
0	No pooling
1	Pooling at the only vallecula
2	Pooling in vallecula and piriform sinus and no penetration into larynx
3	Pooling in vallecula and piriform sinus and penetration into larynx
(2) The glottal closure reflex induced by touching the epiglottis or arytenoid with the endoscope	
0	Marked reflex by one touching
1	Slow and/or weak reflex by one touching
2	Reflex by two or three touching
3	No reflex despite three touching
(3) The location of the bolus at the swallowing reflex initiation assessed by “white-out” timing	
0	Pharyngeal
1	Vallecula
2	Piriform sinuses
3	No swallowing
(4) The extent of pharyngeal clearance after blue-dyed water is swallowed	
0	No residue
1	Pharyngeal residues remain, but are absent after swallowing is attempted two or three times
2	Pharyngeal residues remain, but no penetration into larynx
3	Pharyngeal residues remain, and penetration into larynx

Evaluation of FOSS

The degree of dysphagia was evaluated using the FOSS six months after surgery. The FOSS categorizes swallowing function into six stages as follows: stage 0, normal function and asymptomatic; stage 1, normal function with episodic or daily symptoms of dysphagia; stage 2, compensated abnormal function manifested by significant dietary modifications or prolonged mealtimes but without weight loss or aspiration; stage 3, decompensated abnormal function with weight loss of less than 10% of body weight over six months caused by dysphagia or daily coughing, gagging, or aspiration during meals; stage 4, severely decompensated abnormal function with weight loss exceeding 10% of bodyweight over 6 months caused by dysphagia or severe aspiration with bronchopulmonary complications; and stage 5, non-oral feeding for all nutrition [19].

Statistical analysis

The Kruskal­-Wallis test was used to compare the mean rank of each timepoint. Dunn’s test was employed for multiple comparisons of preoperation versus postoperative timepoints. Logistic regression analysis was performed to explore the factors that impaired Hyodo scores. Statistical tests were performed using GraphPad Prism 9.5.1 (Graph Pad Software, Boston, MA, USA), and logistic regression analysis was performed using the R-powered data tool Exploratory v6.12.5 (Exploratory Inc., Mill Valley, CA). Statistical significance was established at p < 0.05.

Ethical statement

Every participant in this study gave their informed consent for inclusion. The research adhered to the principles of the Declaration of Helsinki, and the protocol received approval from the institutional review board (Tokai University, 08-R98).

## Results

Patient characteristics

This study included 73 patients (59 males and 14 females) with 79 lesions and a median age of 71 years. The median BMI was 20.845 kg/m^2^.

There were nine primary lesions in the supraglottis (four in the epiglottis, five in the arytenoid), 38 in the hypopharynx (25 in the piriform sinus, nine in the posterior wall, two post-cricoid, two in the piriform sinus to posterior wall), and 32 in the oropharynx (19 in the tonsils (p16-positive in 14), three in the base of the tongue (all p16-negative), six in the superior wall, one in the lateral wall, three in the posterior wall). Forty-nine lesions were T1, 30 lesions were T2, and six patients had multiple lesions. Fourteen patients had positive cervical lymph nodes and 16 patients underwent neck dissection. There were 35 patients with a history of esophageal cancer (23 with extended resection, 13 with endoscopic resection, five with irradiation), and 20 with a history of head and neck cancer (two with extended resection, four with neck dissection, 12 with transoral resection, six with irradiation). Only two patients underwent temporary tracheostomy to prevent surgically induced airway obstruction, and only two patients received postoperative irradiation. The patient characteristics are summarized in Table 2.

**Table 2:** Patient characteristics All values are presented as frequency unless otherwise noted. IQR; interquartile range, BMI; body mass index.

Age, median (IQR), years			71 (61-74)
Sex			
Male/female			59/14
BMI, median (IQR), kg/m^2^			20.845 (19.05-23.77)
Primary site			
Supraglottis (n=9)	Epiglottis		4
	Arytenoid		5
Hypopharynx (n=38)	Piriform sinus		25
	Posterior wall		9
	Post cricoid		2
	Piriform sinus to posterior wall		2
Orophrynx (n=32)	Tonsil (p16+)		19 (14)
	Base of tongue (p16+)		3 (0)
	Superior wall		6
	Lateral wall		1
	Posterior wall		3
T classification			
T1			49
T2			30
Multiple lesions			6
Lymph node metastasis			14
Neck dissection			16
Tracheostomy			2
Postoperative irradiation			2
Cancer history			
Head and neck cancer (n=20)		Extended resection	2
		Neck dissection	4
		Transoral resection	12
		Irradiation	6
Esophageal cancer (n=35)		Extended resection	23
		Endoscopic resection	13
		Irradiation	5

Outcome of swallowing function

The FOSS score at six months after surgery was grade 0 in 61 patients, grade 1 in 10 patients, and grade 2 in two patients. Most patients were able to consume food orally without any restrictions (Table 3).

**Table 3:** FOSS at six months after surgery FOSS; functional outcome swallowing scale.

FOSS stage	Number of patients
0	61
1	10
2	2

There was no significant worsening of the mean Hyodo score in all subjects. The mean sub-scores also showed no significant difference compared to the preoperative sub-scores (Table 4). However, 23 patients exhibited impaired postoperative Hyodo score. Logistic regression analysis was performed on these 23 impaired patients to estimate predictors using age, BMI, primary site, T classification, neck dissection, history of head and neck cancer, history of radiation therapy to the head and neck, and history of esophageal cancer as predictor variables. Age ≥65 years was a significant predictor (p=0.026, Table 5).

**Table 4:** Score trend of Hyodo score and its subscores SE; standard error.

	Pre-operation (mean±SE)	3 months (mean±SE)	6 months (mean±SE)	12 months (mean±SE)	P-value
Salivary pooling degree at the vallecula and piriform sinuses	0.41±0.067	0.31±0.065	0.38±0.077	0.38±0.112	0.959
Glottal closure reflex induced by touching the epiglottis or arytenoid with endoscope	0.31±0.080	0.33±0.076	0.36±0.081	0.19±0.079	0.746
Location of bolus at swallowing reflex initiation assessed by “white-out” timing	0.53±0.070	0.42±0.074	0.51±0.083	0.31±0.092	0.368
Extent of pharyngeal clearance after swallowing blue-dyed water	0.38±0.069	0.48±0.105	0.35±0.076	0.19±0.148	0.428
Total Hyodo score	1.63±0.196	1.54±0.235	1.53±0.233	1.52±0.256	0.880
number of patients	64	52	53	25	

**Table 5:** Logistic regression analysis to predict Hyodo score impairment. Statistical significance was established at p < 0.05.

Variables	Odds ratio	95% Confidence Interval	P-value
Age			
<65	Reference		
≥65	4.583	1.203-17.454	0.026
Body mass index			
≥18.5	Reference		
<18.5	1.246	0.296-5.236	0.764
Primary lesion			
Supraglottis/ Hypopharynx	Reference		
Oropharynx	0.62	0.151-2.547	0.507
T classification			
T1	Reference		
T2	0.836	0.193-3.627	0.811
Neck Dissection			
No	Reference		
Yes	1.121	0.209-6.007	0.894
History of head and neck cancer			
No	Reference		
Yes	2.023	0.371-11.026	0.416
Irradiation history for head and neck			
No	Reference		
Yes	0.59	0.048-7.269	0.68
History of esophageal cancer			
No	Reference		
Yes	0.265	0.055-1.272	0.097

In the time series, the Hyodo score of the impaired patient group significantly worsened at three and six months postoperatively (p=0.0002 and p=0.008, respectively; Table 6). Sub-scores also showed worsening at three and six months postoperatively for the glottal closure reflex induced by touching the epiglottis or arytenoid with the endoscope (p=0.0015, p=0.0374) and at three months postoperatively for the extent of pharyngeal clearance after blue-dyed water was swallowed (p=0.0196). No significant differences were found in other parameters such as the salivary pooling degree at the vallecula and piriform sinuses and the location of the bolus at the swallowing reflex initiation assessed by “white-out” timing.

**Table 6:** Score detail of Hyodo score and sub-score of impaired patients. The Kruskal­-Wallis test was used to compare the mean rank of each time point. Dunn’s test was employed for multiple comparisons of preoperation versus postoperative timepoints. Statistical significance was established at p < 0.05. SE; Standard error

	Pre operation (mean±SE)	3months (mean±SE; P-value)	6months (mean±SE; P-value)	12months (mean±SE; P-value)	P-value
1. The salivary pooling degree at the vallecula and piriform sinuses	0.348±0.102	0.619±0.011; 0.2579	0.571±0.130; 0.6134	0.636±0.203; 0.6086	0.321
2. The glottal closure reflex induced by touching the epiglottis or arytenoid with the endoscope	0.043±0.043	0.619±0.146; .0015	0.477±0.148; 0.0374	0.182±0.122; >0.9999	0.003
3. The location of the bolus at the swallowing reflex initiation assessed by “white-out” timing	0.435±0.123	0.762±0.118; 0.1291	0.714±0.122; 0.2524	0.364±0.152; >0.9999	0.076
4. The extent of pharyngeal clearance after blue-dyed water is swallowed	0.214±0.088	0.857±0.199; 0.0196	0.524±0.131; 0.3147	0.727±0.237; 0.1487	0.039
Hyodo Score	1.043±0.231	2.857±0.367; 0.0002	2.286±0.332; 0.008	1.909±0.415; 0.2286	0.0005
number of patients	23	21	21	11	

Outcome of QOL

**Figure 1:**
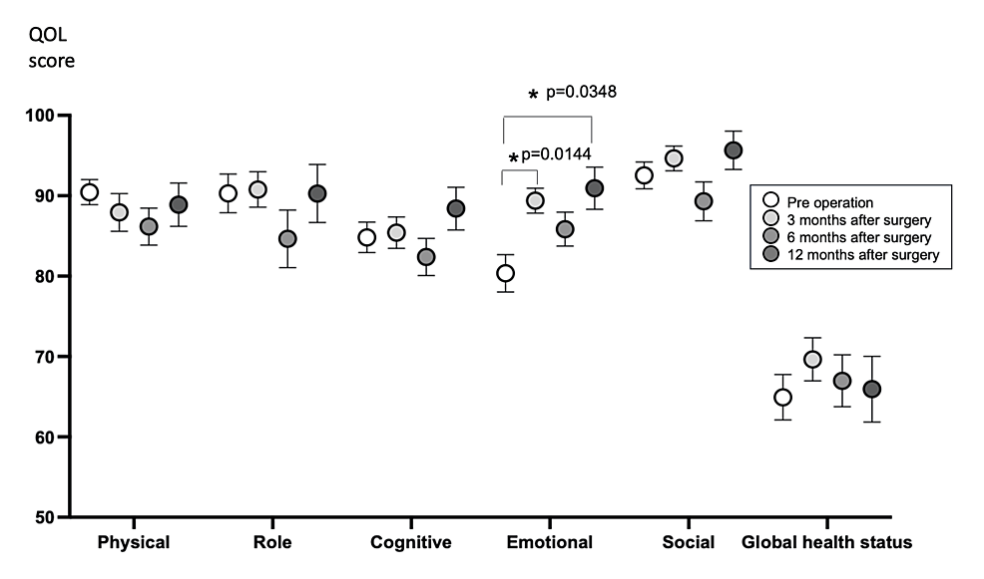
Score trend of EORTC QLQ C30 Functional scales. Multiple comparisons of preoperation versus postoperative time points, Statistical significance was established at p < 0.05 (Asterisk).

Changes in EORTC QLQ C30 Functional Scale scores are shown in Figure 1. In the EORTC QLQ C30 Functional Scale, a higher score is interpreted as a better QOL. The Kruskal-Wallis test showed significant improvement in emotional score (p=0.016), which were significantly better at three months (p=0.0144) and 12 months (p=0.0348) after surgery compared to preoperation. No significant differences were observed among the other parameters.

**Figure 2:**
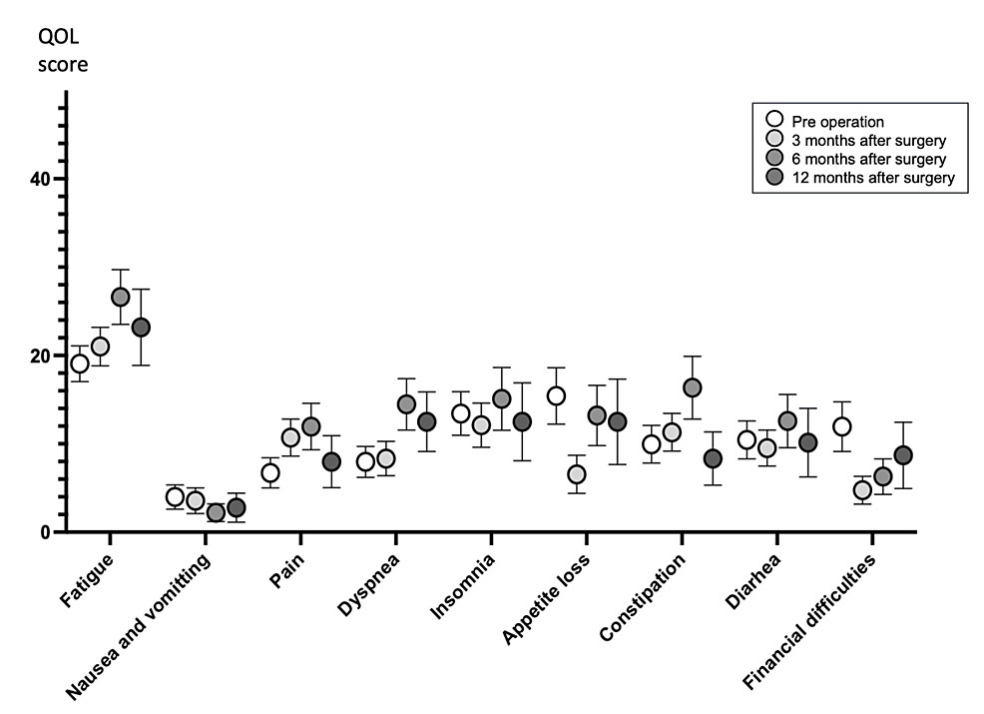
Score trend of EORTC QLQ C30 symptom scales. Multiple comparisons of preoperation versus postoperative time points, Statistical significance was established at p < 0.05.

Changes in EORTC QLQ C30 Symptom Scale scores are shown in Figure 2. In contrast to the functional scales, the lower the score on the symptom scales, the higher the QOL. The Kruskal-Wallis test showed no significant differences for any of the symptom scale parameters based on the measurement time points.

**Figure 3:**
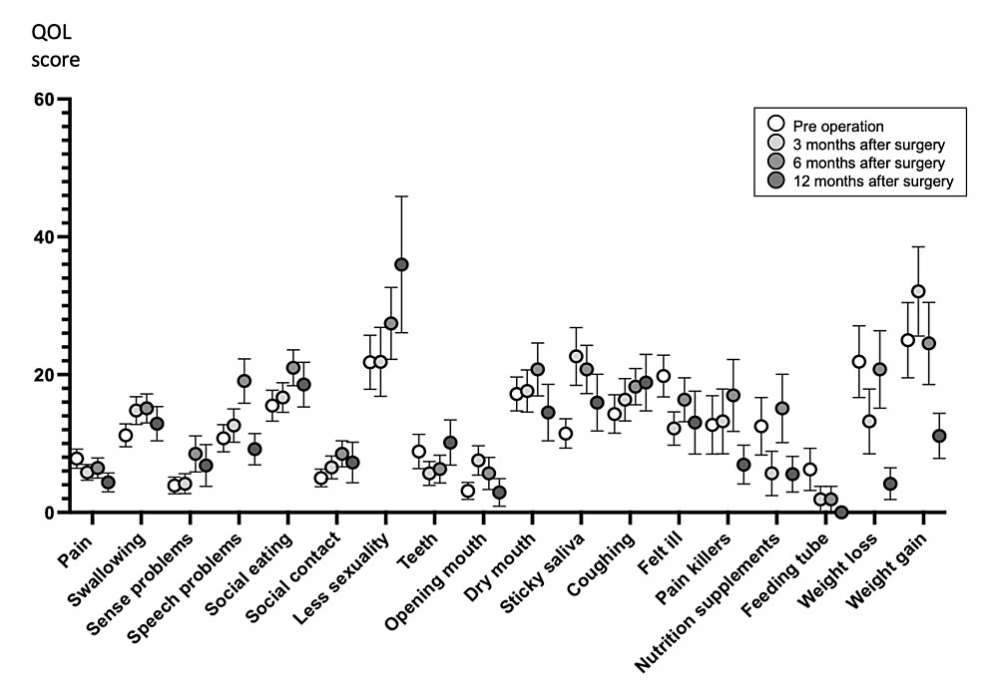
Score trend of EORTC QLQ H&N35. Multiple comparisons of preoperation versus postoperative time points, Statistical significance was established at p < 0.05.

Changes in EORTC QLQ H&N35 scores are shown in Figure 3. As with the EORTC QLQ C30 Symptom Scales, the smaller score indicates a higher QOL for H&N35. The Kruskal-Wallis test showed no significant differences for any of its respective parameters based on the measurement time points. No significant deterioration in QOL was identified after TOVS in either questionnaire method.

## Discussion

The mean QOL scores showed no significant differences before and after TOVS. In addition, the FOSS at six months after surgery showed that the overall level of oral intake was maintained, suggesting that TOVS is useful in maintaining swallowing function.

Neither the total Hyodo score, nor its sub-scores worsened overall, but by examining only exacerbation cases, we found exacerbation factors. In previous reports, predictors of dysphagia included ECOG PS, age ≥80 years, low BMI, and a history of head and neck irradiation [24-26]. We selected age, BMI, history of head and neck cancer, and history of head and neck irradiation as predictors for multivariate analysis. The age cut-off was 65 years, which is the World Health Organization (WHO) standard for elderly adults, and the BMI cut-off was 18.5 kg/m^2^, which is also the WHO standard for underweight. In addition, because of ADLH2 gene polymorphisms, esophageal squamous cell carcinoma is an important overlapping cancer of the head and neck region in Japan [8]; therefore, a history of esophageal cancer was added as a predictive factor. Neck dissection was also considered because surgical manipulation of the neck might affect swallowing function. The ECOG PS was not considered as all patients had an ECOG PS of 0. Consequently, age ≥65 years was the only predictor of a worsening Hyodo score.

The trend of the Hyodo score of impaired patients suggests that the score deterioration peaks at three and six months postoperatively, especially the glottal closure reflex induced by touching the epiglottis or arytenoid with the endoscope and the extent of pharyngeal clearance after blue-dyed water is swallowed. The trend is similar to other TORS reports on postoperative swallowing that have shown improvement over time [16]. Fujiwara et al. showed that the postoperative FOSS was worse in patients with aspiration on preoperative videofluoroscopy [24]. Although this study did not include cases of obvious aspiration on preoperative FEES, postoperative dysphagia due to decreased laryngeal perception and poor pharyngeal clearance should be considered in elderly patients.

One report showed improvement in endoscopic swallowing scores in elderly patients with dysphagia after laryngeal elevation training such as the swallowing forehead exercise and the chin push-pull maneuver without highly loaded head lifting [27]. Therefore, the introduction of perioperative rehabilitation may contribute to the maintenance or early recovery of swallowing function after transoral resection. Further studies are warranted.

There are reports of good QOL after microscopic mucosal resection [28], good voice and swallowing function after ELPS [13], and good QOL after transoral laser microsurgery for hypopharyngeal cancer [29]. Half of the patients in this study also had hypopharyngeal cancer, and despite differences in conventional surgical and evaluation methods, transoral resection for early stage pharyngolaryngeal cancer may contribute to the maintenance of QOL.

Some reports on transoral resection and QOL after TORS have been published, mostly in patients with oropharyngeal cancer. The ORATOR study reported worse QOL in the TORS + neck dissection group than in the radiation group [18]. However, some studies have suggested that transoral resection maintains the patient’s QOL [15,16]. In the ORATOR study, >70% of patients received postoperative radiotherapy after TORS. In the other studies, TORS alone or postoperative irradiation was used in less than half of all cases. The present study was conducted in a group of patients where only a few received postoperative irradiation, and our results show that QOL was maintained. Postoperative irradiation has a significant impact on QOL; therefore, the key to maintaining QOL is selecting patients who do not require postoperative irradiation. A detailed preoperative study of resectability is required to ensure successful transoral resection with a preferable QOL.

This study had several limitations. Although this was a prospective study, it was conducted at a single institution. This was not a comparison with other treatments, and a selection bias exists. The study also had many missing statistical values and was not a complete study of repeat cases. The Hyodo Score is simple, and widely used in Japanese clinical practice. The Japanese version is easily available and, therefore used in this study instead of the other validated FEES scales such as the penetration-aspiration scale [30], it should be noted that the Hyodo score is an unconventional evaluation method. Furthermore, many of this population have previous history of esophageal cancer and other head and neck cancers. They may be distinct from other populations; therefore, the study results may not be generalizable. However, quite favorable swallowing function and QOL are still given at the tumor sites.

## Conclusions

TOVS generally preserves good swallowing function. However, there have been cases of deterioration of swallowing function peaking at three to six months postoperatively. Particular attention should be paid to the decline in swallowing function due to laryngeal imperception and poor pharyngeal clearance, especially in the elderly over 65 years of age.

QOL after TOVS is well maintained in the group of patients with no postoperative irradiation. Careful selection of patients whose treatment can be completed with transoral resection alone will help maintain QOL.

## References

[1] Forastiere AA, Goepfert H, Maor M (2003). Concurrent chemotherapy and radiotherapy for organ preservation in advanced laryngeal cancer. N Engl J Med.

[2] Machtay M, Moughan J, Trotti A (2008). Factors associated with severe late toxicity after concurrent chemoradiation for locally advanced head and neck cancer: an RTOG analysis. J Clin Oncol.

[3] Forastiere AA, Zhang Q, Weber RS (2013). Long-term results of RTOG 91-11: a comparison of three nonsurgical treatment strategies to preserve the larynx in patients with locally advanced larynx cancer. J Clin Oncol.

[4] O'Malley BW Jr, Weinstein GS, Snyder W, Hockstein NG (2006). Transoral robotic surgery (TORS) for base of tongue neoplasms. Laryngoscope.

[5] Weinstein GS, O'Malley BW Jr, Snyder W, Sherman E, Quon H (2007). Transoral robotic surgery: radical tonsillectomy. Arch Otolaryngol Head Neck Surg.

[6] Chaturvedi AK, Engels EA, Pfeiffer RM (2011). Human papillomavirus and rising oropharyngeal cancer incidence in the United States. J Clin Oncol.

[7] Cracchiolo JR, Baxi SS, Morris LG, Ganly I, Patel SG, Cohen MA, Roman BR (2016). Increase in primary surgical treatment of T1 and T2 oropharyngeal squamous cell carcinoma and rates of adverse pathologic features: National Cancer Data Base. Cancer.

[8] Katada C, Muto M, Nakayama M (2012). Risk of superficial squamous cell carcinoma developing in the head and neck region in patients with esophageal squamous cell carcinoma. Laryngoscope.

[9] Muto M, Nakane M, Katada C (2004). Squamous cell carcinoma in situ at oropharyngeal and hypopharyngeal mucosal sites. Cancer.

[10] Satou Y, Omori T, Tagawa M (2006). Treatment of superficial carcinoma in the hypopharynx (Article in Japanese). Nihon Jibiinkoka Gakkai Kaiho.

[11] Shiotani A, Tomifuji M, Araki K, Yamashita T, Saito K (2010). Videolaryngoscopic transoral en bloc resection of supraglottic and hypopharyngeal cancers using laparoscopic surgical instruments. Ann Otol Rhinol Laryngol.

[12] Imanishi Y, Ozawa H, Sakamoto K (2017). Clinical outcomes of transoral videolaryngoscopic surgery for hypopharyngeal and supraglottic cancer. BMC Cancer.

[13] Tateya I, Muto M, Morita S (2016). Endoscopic laryngo-pharyngeal surgery for superficial laryngo-pharyngeal cancer. Surg Endosc.

[14] Tomifuji M, Araki K, Yamashita T, Shiotani A (2014). Transoral videolaryngoscopic surgery for oropharyngeal, hypopharyngeal, and supraglottic cancer. Eur Arch Otorhinolaryngol.

[15] Choby GW, Kim J, Ling DC (2015). Transoral robotic surgery alone for oropharyngeal cancer: quality-of-life outcomes. JAMA Otolaryngol Head Neck Surg.

[16] Meulemans J, Vanermen M, Goeleven A (2022). Transoral robotic surgery (TORS) using the da Vinci Xi: prospective analysis of feasibility, safety, and outcomes. Head Neck.

[17] Tomifuji M, Araki K, Uno K (2020). Transoral videolaryngoscopic surgery for laryngeal and hypopharyngeal cancer - technical updates and long-term results. Auris Nasus Larynx.

[18] Nichols AC, Theurer J, Prisman E (2019). Radiotherapy versus transoral robotic surgery and neck dissection for oropharyngeal squamous cell carcinoma (ORATOR): an open-label, phase 2, randomised trial. Lancet Oncol.

[19] Salassa JR (1999). A functional outcome swallowing scale for staging oropharyngeal dysphagia. Dig Dis.

[20] Chiba Y, Sano D, Ikui Y (2018). Predictive value of the Hyodo score in endoscopic evaluation of aspiration during swallowing. Auris Nasus Larynx.

[21] Aaronson NK, Ahmedzai S, Bergman B (1993). The European Organization for research and treatment of cancer QLQ-C30: a quality-of-life instrument for use in international clinical trials in oncology. J Natl Cancer Inst.

[22] Toth G, Tsukuda M (2004). The European Organisation for Research and Treatment of Cancer (EORTC) Quality of Life Questionnaire for Japanese patients with head and neck cancer--the Japanese version of QLQ-H&amp;N35 (Article in Japanese). Gan To Kagaku Ryoho.

[23] Watanabe Y, Tanaka S, Hiratsuka Y (2020). Defect repair with fibrin glue/polyglycolic acid after endoscopic laryngopharyngeal cancer resection. Laryngoscope.

[24] Fujiwara K, Taira K, Donishi R, Koyama S, Morisaki T, Fukuhara T, Takeuchi H (2021). Preoperative predictors of dysphagia after transoral surgery. Int J Clin Oncol.

[25] Tomifuji M, Araki K, Yamashita T (2016). Risk factors for dysphagia after transoral videolaryngoscopic surgery for laryngeal and pharyngeal cancer. Head Neck.

[26] Ueda T, Yumii K, Urabe Y (2022). Swallowing function after transoral surgery for laryngopharyngeal cancer. PLoS One.

[27] Sugaya N, Goto F, Seino Y, Nishiyama K, Okami K (2021). The effect of laryngeal elevation training on swallowing function in patients with dysphagia. J Laryngol Otol.

[28] Okami K, Ebisumoto K, Sakai A (2013). Transoral en bloc resection of superficial laryngeal and pharyngeal cancers. Head Neck.

[29] Hung LT, Huang HI, Wang LW, Yang MH, Chu PY (2018). Oncologic results and quality of life in patients with squamous cell carcinoma of hypopharynx after transoral laser microsurgery. Lasers Surg Med.

[30] Robbins J, Coyle J, Rosenbek J, Roecker E, Wood J (1999). Differentiation of normal and abnormal airway protection during swallowing using the penetration-aspiration scale. Dysphagia.

